# Case report: Dual atrioventricular nodal non-reentrant tachycardia with six types of ECG patterns leading to tachycardia-induced cardiomyopathy in a 51-year-old man

**DOI:** 10.3389/fcvm.2022.998453

**Published:** 2022-10-12

**Authors:** Man-Yi Ren, Yong Zhang, Yu-Jiao Zhang, Mei Gao, Cai-Hua Sang, Yong-Mei Cha, Ying-Long Hou

**Affiliations:** ^1^Shandong Medicine and Health Key Laboratory of Cardiac Electrophysiology and Arrhythmia, Department of Cardiology, The First Affiliated Hospital of Shandong First Medical University & Shandong Provincial Qianfoshan Hospital, Jinan, China; ^2^Department of Cardiology, Beijing Anzhen Hospital, National Clinical Research Centre for Cardiovascular Diseases, Capital Medical University, Beijing, China; ^3^Division of Electrophysiology, Department of Cardiovascular Diseases, Mayo Clinic, Rochester, MN, United States

**Keywords:** dual atrioventricular nodal non-reentrant tachycardia, electrocardiogram, cardiomyopathy, slow pathway, catheter ablation

## Abstract

More than three types of ECG manifestations in one patient with dual atrioventricular nodal non-reentrant tachycardia (DAVNNT) are rare. We report a 51-year-old male patient with DAVNNT consisting of six types of ECG patterns leading to tachycardia-induced cardiomyopathy. After radiofrequency ablation of the slow pathway, DAVNNT was eliminated and cardiac function was restored.

## Introduction

Dual atrioventricular (AV) nodal non-reentrant tachycardia (DAVNNT) is characterized by a sinus beat resulting in consecutive double antegrade conduction via the fast and slow pathways, which produces two ventricular depolarizations (1). The 12-lead surface ECG has been regarded as the gold standard for the diagnosis of DAVNNT with “2 QRS for 1 P” manifestation (2). However, apart from the typical ECG pattern with 1:2 AV conduction over the fast and slow pathways (3–12), there are six atypical types of ECG patterns including either slow or fast pathway antegrade block (3, 4, 6, 7), both fast and slow pathway block (4, 5), alternating pathway block (3, 4), functional bundle branch block (4, 10), and atrioventricular nodal reentry tachycardia (AVNRT) (3, 10) during 1:2 AV conduction. Additionally, DAVNNT could result in tachycardia-induced cardiomyopathy (TIC) (2, 6, 10). Nevertheless, more than three types of ECG manifestations in one patient are rare. Herein, we report a case with DAVNNT consisting of six types of ECG patterns leading to TIC.

## Case description

**FIGURE 1:**
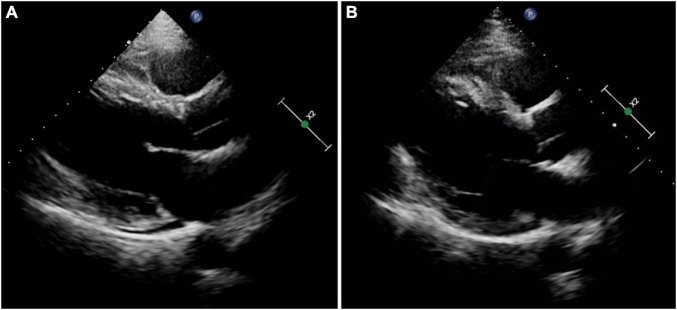
Results of transthoracic echocardiography. **(A)** On admission. **(B)** At the 3-month follow-up.

**FIGURE 2:**
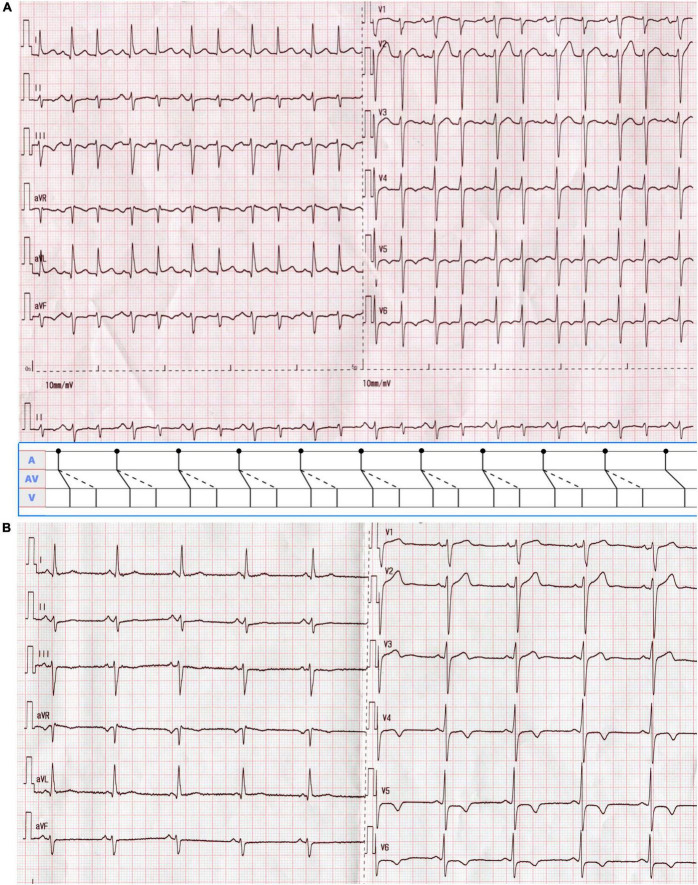
**(A)** A 12-lead ECG on admission showed supraventricular tachycardia with alternating RR intervals at a rate of 133 beats/min. The P wave was positive in lead II, negative in aVR, and biphasic in V_1_, suggesting a sinus rhythm of 66 beats/min. Each sinus beat was simultaneously conducted through the anterograde fast and slow AV nodal pathways, giving rise to a double ventricular response. QRS alternans were present as the first and second QRS complexes during 1:2 AV conduction were of different amplitudes. As shown in the ladder diagram, dots indicate the beginnings of the P waves. Solid oblique and dashed lines in the AV portion of the diagram denote conduction over the fast and slow pathways, respectively. **(B)** The surface ECG returned to normal after radiofrequency ablation.

A 51-year-old man was presented with palpitations for 9 years and had a documented history of premature atrial and ventricular contractions and paroxysmal atrial fibrillation. He was admitted for incessant narrow complex tachycardia and New York Heart Association class III heart failure. He took perindopril 4 mg twice daily, metoprolol succinate 23.75 mg once daily, furosemide 20 mg every other day, and aldosterone 20 mg every other day for half a month, which failed to alleviate his symptoms. His physical examination and laboratory tests were unremarkable. Computed tomography angiography excluded coronary artery disease. Transthoracic echocardiography (TTE) revealed a dilated cardiomyopathy with global hypokinesis, left ventricular end-diastolic diameter (LVEDD) 68 mm, and left ventricular ejection fraction (LVEF) 39% (Figure 1A). A 12-lead ECG showed a narrow complex tachycardia with 510 and 400 msec alternating R-R intervals. Each P wave was followed by 2 QRS, suggesting 1:2 AV conduction through the fast and slow AV nodal pathways and possible DAVNNT (Figure 2A).

**FIGURE 3:**
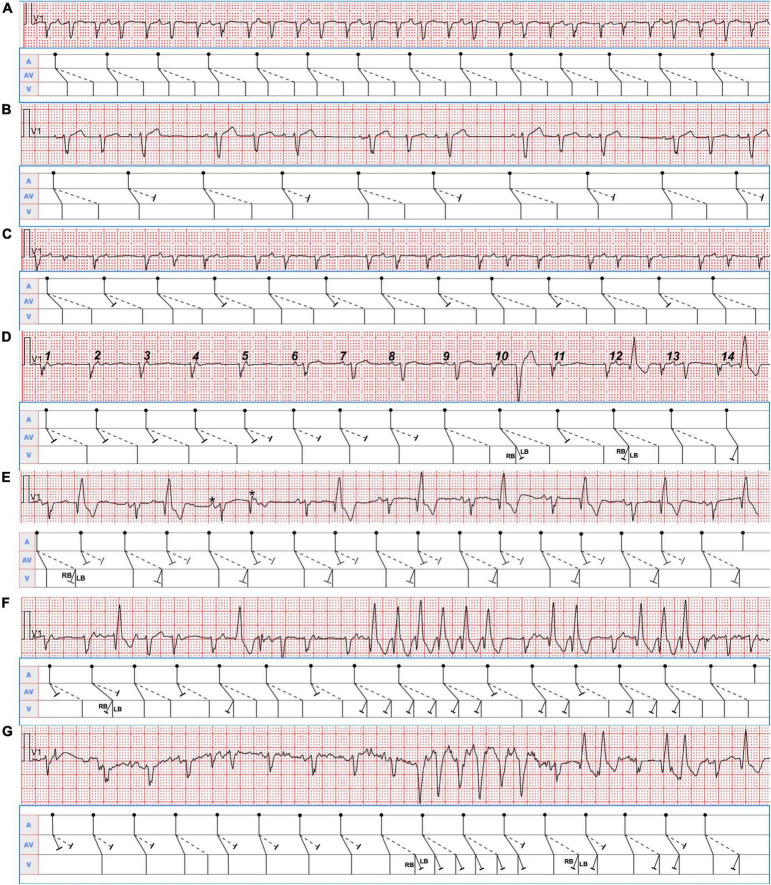
Seven typical Holter recording strips in lead V_1_ were shown. The corresponding mechanism was explained in the ladder diagram below each of them. RB, right bundle; LB, left bundle. **(A)** The sinus P waves (PP intervals 740 msec) were conducted simultaneously over the dual AV nodal pathways, producing a double ventricular response. **(B)** Group beatings with PP intervals of 1,120 msec were presented. A group of three was seen throughout the tracing follow by a pause, suggesting sinus beats conducted with alternating 1:2 and 1:1 AV ratios with longer PR intervals (160 msec) during 1:1 conduction over the fast pathways. **(C)** The sinus beats (PP intervals 820 msec) in the group beatings conducted with alternating 1:1 and 1:2 AV ratios with shorter PR intervals (580 msec) during 1:1 conduction over the slow pathways. **(D)** The front 5 and eleventh P waves were conducted only through the slow pathways, then the sixth to eighth P waves only *via* the fast pathways, and the ninth, tenth, twelfth, and thirteenth P waves simultaneously over the dual AV pathways. The tenth P wave with a PR interval of 240 msec gave rise to a QRS complex with left bundle branch block (LBBB) morphology, whereas the twelfth and fourteenth P waves with a PR interval of 220 msec produced a QRS wave with right bundle block (RBBB) pattern. The front 7 PP intervals were 720 msec, whereas the remaining PP intervals were approximately 840 msec. **(E)** The remaining adjacent PP intervals will be determined according to the PP interval of 600 msec consisting of two P waves marked with asterisks. The 2:1 conduction block occurred when the sinus beats were conducted simultaneously *via* the fast and slow pathways. Additionally, RBBB aberrancy except the sixth QRS complex were presented with conduction through the slow pathways. **(F)** Single, double, triple, and especially six consecutive QRS complexes with RBBB configuration from the conduction *via* the fast and slow pathways were shown in the lead V_1_ rhythm strip. It was the most difficult to identify them due to the superimposition of the P waves (PP intervals 640 msec) on the QRS complexes. **(G)** As a result of the conduction *via* the fast and (or) slow pathways, single and double QRS complexes with RBBB pattern as well as six consecutive QRS complexes with LBBB morphology coexisted in the lead V_1_ rhythm strip. Likewise, the P waves with PP intervals of 620 msec were superimposed on the QRS complexes when LBBB or RBBB occurred, which made them unrecognizable.

Different patterns of AV conduction recorded from Holter monitoring were displayed in Figure 3. Pattern 1 showed a continuous manifest 1:2 AV conduction over the fast and slow pathways (Figure 3A). Pattern 2 was defined as the slow pathway with a 2:1 conduction block (Figure 3B). Pattern 3 occurred when the conduction of the fast pathway was intermittently blocked (2:1) (Figure 3C). Pattern 4 was classified as the alternating conduction block of the fast and slow pathways (Figure 3D). Pattern 5 was typified by the simultaneous antegrade 2:1 conduction block with the dual AV pathways (Figure 3E). In pattern 6, there was 1:2 conduction with persistent or intermittent left or right bundle branch block (Figures 3F,G).

**FIGURE 4:**
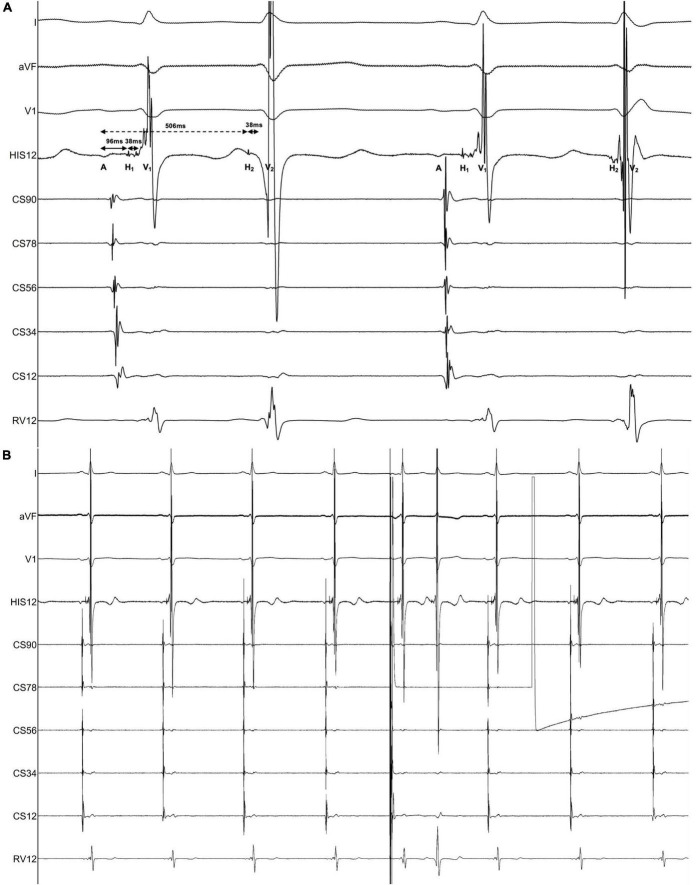
Findings of the electrophysiological study. The intracardiac recording confirmed the diagnosis of dual AV nodal non-reentrant tachycardia (DAVNNT). **(A)** One atrial impulse (A) triggered one hissian potential (H_1_) and one ventricular potential (V_1_) through the fast pathway, and then one H_2_ and one V_2_ through the slow pathway. The time difference between the fast pathway with an AH interval of 96 msec and the slow pathway with an AH interval of 506 msec was long enough to result in a double ventricular response. The HV interval of both fast and slow pathways was 38 msec. This recording was taken at a sweep speed of 100 mm/s. **(B)** Atrial extra systolic stimulation can reproducibly induce a 1:2 conduction over the fast and slow pathways, corroborating the diagnosis of DAVNNT. A paper speed was 25 mm/s.

The electrophysiological study (EPS) revealed that each sinus beat was conducted down both the fast and slow pathways, resulting in 2 QRS, which could be reproduced by atrial extra stimulus (Figure 4). Atrial programmed extra stimulation at CS_7,8_ was performed using a driving cycle length (S_1_) of 600 msec. When the S_1_S_2_ interval was reduced to 350 msec, a marked AH jump from 172 to 328 msec was observed, which did not trigger an atrial echo and AVNRT even with intravenous isoprenaline. There was no retrograde ventriculoatrial (VA) conduction. Radiofrequency ablation of the slow pathway subsequently eliminated the tachycardia and restored 1:1 AV conduction (Figure 2B). Oral medications except metoprolol succinate 11.875 mg once daily after discharge was the same as at admission. At the 3-month follow-up, the patient was free of symptoms. Holter recordings showed no recurrent 1:2 AV conduction. LVEF improved to 67% and the LVEDD decreased to 56 mm (Figure 1B).

## Discussion

In 1975, the dual ventricular response was first described as a manifestation of dual AV nodal physiology in which a premature atrial depolarization was conducted down both the fast and slow pathways, called “double fire” (9) and subsequently named DAVNNT (12). The surface ECG characterizes a sinus P wave followed by two narrow QRS complexes (2). However, when the frequency of the sinus P wave is over 100 bpm, the ventricular rate will amount to 200 bpm or more. Meanwhile, the sinus P waves could be superimposed on the preceding QRS complexes, T waves, and ST segments, making DAVNNT diagnosis difficult (Figures 3E–G). Hence, DAVNNT is often misdiagnosed as premature atrial or ventricular complexes, supraventricular tachycardia, atrial fibrillation, and ventricular tachycardia, leading to inappropriate referral for pulmonary vein isolation (11) and improper implantation of a pacemaker (7). A misdiagnosis (1) may result in TIC, as in our case, for 9 years.

Seven ECG patterns of DAVNNT from different patients have been reported. More than three ECG manifestations in one patient are rare (3, 4). Our case showed six different ECG presentations. The variable ECG manifestations are attributable to the fact that dual AV nodal pathways have distinct refractory periods, different conduction velocities, Wenckebach-type block, and unidirectional block (3), and various degrees of concealed retrograde conduction under different physiologic states (7). Specifically, in Figure 3B, slow pathway conduction prolonged the following fast pathway conduction by retrograde concealed penetration of the fast pathway, which made the ensuing slow pathway conduction blocked due to concealed conduction. As the second P wave did not conduct down the slow pathway, there was no retrograde concealed conduction to the fast pathway, elucidating the short fast pathway conduction following the third P wave. Similarly, in Figure 3C, slow pathway conduction blocked the following fast pathway conduction by retrograde concealed penetration of the fast pathway, making the second PR interval of slow pathway conduction 80 msec shorter compared with the first PR interval. In Figure 3D, after Mobitz type I AV block (Wenckebach) conduction in the front 5 P waves occurred only along with slow pathway, the fast pathway exhibited Wenckebach conduction in the sixth to eleventh P waves. In Figure 3E, the third PR interval down the slow pathway was 20 msec longer than the remaining PR intervals, so the fifth P wave can conduct through the slow pathway and produce a normal QRS complex. In Figures 3F,G, the QRS complexes displayed functional RBBB and LBBB morphologies because of different PP intervals (rate-dependent aberrancy), retrograde concealed conductions, and changes in autonomic nervous tension. Taken together, different sinus rates led to different manifestations of DAVNNT. Therefore, knowing all of the six ECG presentations of DAVNNT will help early diagnosis and management.

The differential diagnosis of 12-lead ECG presentation, in this case, included (1) orthodromic atrioventricular reentry tachycardia (AVRT) with alternating AV conduction over a slow and a fast AV nodal pathway as well as VA conduction over an accessory pathway, (2) AVNRT with reentrant circuit using two distinct, beat-to-beat alternating slow AV nodal pathways anterogradely and a single fast pathway retrogradely, (3) atrial bigeminy, and (4) junctional bigeminy with a retrograde conduction block. However, the regular presence of sinus P waves excluded both AVRT and AVNRT. Additionally, atrial bigeminy can be ruled out due to the absence of atrial P waves preceding the QRS complexes with a short cycle length. In addition, some typical rhythm strips in lead V_1_ from Holter ECG tracings (Figure 3) demonstrated that each sinus P wave was conducted simultaneously over the fast and slow pathways, and produced two QRS complexes, which were further corroborated by EPS. Remarkably, the antegrade fast pathway is more sensitive to the effects of adenosine than the slow pathway. A positive adenosine test verified by a PR jump can identify dual AV node physiology on surface ECG recording (13).

No medications appear highly successful in suppressing DAVNNT. Radiofrequency ablation or cryoablation of the slow pathway can eliminate DAVNNT and restore left ventricular function (1).

## Limitations

The Holter tracings in Figures 3B–G were not verified by EPS, which did not influence the correct explanations in the ladder diagrams.

## Conclusion

This is the first case that reveals six ECG patterns of DAVNNT leading to TIC. Radiofrequency ablation of the slow pathway eliminated DAVNNT and restored cardiac function.

## Data availability statement

The raw data supporting the conclusions of this article will be made available by the authors, without undue reservation.

## Ethics statement

The studies involving human participants were reviewed and approved by the Ethics Committee of the First Affiliated Hospital of Shandong First Medical University. The patients/participants provided their written informed consent to participate in this study and for the publication of this case report.

## Author contributions

M-YR, YZ, Y-JZ, and C-HS contributed to the clinical treatment of this case. M-YR wrote the manuscript. Y-MC, Y-LH, and MG revised the manuscript. All authors contributed to the article and approved the submitted version.
